# Dissemination of Evidence-Based Recommendations for Sickle Cell Disease to Primary Care and Emergency Department Providers in North Carolina: A Cost Benefit Analysis

**DOI:** 10.36469/jheor.2021.21535

**Published:** 2021-04-01

**Authors:** Paula Tanabe, Audrey L. Blewer, Emily Bonnabeau, Hayden B. Bosworth, Denise H. Clayton, Nancy Crego, Marian F. Earls, Kern Eason, Grayson Forlines, Gary Rains, Matthew Young, Nirmish Shah

**Affiliations:** 1 School of Nursing, Duke University, Durham, NC, USA; Duke University School of Medicine, Durham, NC, USA; 2 Department of Family Medicine and Community Health, School of Medicine, Duke University, Durham, NC, USA; Department of Population Health Sciences, School of Medicine, Duke University, Durham, NC, USA; 3 School of Nursing, Duke University, Durham, NC, USA; 4 School of Nursing, Duke University, Durham, NC, USA; Duke University School of Medicine, Durham, NC, USA; Department of Population Health Sciences, School of Medicine, Duke University, Durham, NC, USA; Center of Innovation to Accelerate Discovery and Practice Transformation (ADAPT), Durham Veterans Affairs Medical Center (DVAMC), Durham, NC, USA; 5 RTI International, NC, USA; 6 Marian F. Earls Consulting, LLC, Greensboro, NC, USA; 7 Previously – Community Care North Carolina, Cary, NC, USA; 8 Duke University School of Medicine, Durham, NC, USA; 9 Wake Emergency Physicians P.A., Raleigh, NC, USA

**Keywords:** toolkit, dissemination, cost benefit analysis, sickle cell

## Abstract

**Background:** Sickle cell disease (SCD) is a genetic condition affecting primarily individuals of African descent, who happen to be disproportionately impacted by poverty and who lack access to health care. Individuals with SCD are at high likelihood of high acute care utilization and chronic pain episodes. The multiple complications seen in SCD contribute to significant morbidity and premature mortality, as well as substantial costs to the healthcare system.

**Objectives:** SCD is a complex chronic disease resulting in the need for primary, specialty and emergency care. Many providers do not feel prepared to care for individuals with SCD, despite the existence of evidence-based guidelines. We report the development of a SCD toolbox and the dissemination process to primary care and emergency department (ED) providers in North Carolina (NC). We report the effect of this dissemination on health-care utilization, cost of care, and overall cost-benefit.

**Methods:** The SCD toolbox was adapted from the National Heart, Lung, and Blood Institute recommendations. Toolbox training was provided to quality improvement specialists who then disseminated the toolbox to primary care providers (PCPs) affiliated with the only NC managed care coordination system and ED providers. Tools were made available in paper, online, and in app formats to participating managed care network practices (n=1 800). Medicaid claims data were analyzed for total costs and benefits of the toolbox dissemination for a 24-month pre- and 18-month post-intervention period.

**Results:** There was no statistically significant shift in the number of outpatient specialty visits, ED visits or hospitalizations. There was a small decrease in the number of PCP visits in the post-implementation period. The dissemination resulted in a net cost-savings of 361 414(14.03 per-enrollee per-month on average). However, the estimated financial benefit associated with the dissemination of the SCD toolbox was not statistically significant.

**Conclusions:** Although we did not find the expected shift to increased PCP visits and decreased ED visits and hospitalizations, there were many lessons learned.

## BACKGROUND

Sickle cell disease (SCD) is a genetic condition affecting 100 000 individuals in the United States; this group is primarily composed of individuals of African descent, who happen to be disproportionately impacted by poverty and poor access to health care.^1^ Individuals with SCD are at high likelihood to develop chronic organ damage and experience pain frequently, leading to high acute care utilization (emergency room visits and hospitalizations).^1,2^ Some individuals with SCD experience both acute and chronic pain, requiring the use of opioids. To prevent the occurrence of these frequently experienced pain episodes referred to as vaso-occlusive crisis (VOC), hydroxyurea (HU) is often prescribed.^3^ The multiple complications seen in SCD contribute to significant morbidity and premature mortality, as well as substantial costs to the health-care system. In a recent analysis of mortality trends in SCD from 1979–2017, the median age of death increased from 28 in 1979 to 43 in 2016. However, the overall mean age of death in the United States in 2016 was 75, and among those with SCD it was 40.^4^ In the most recent available data, in 2016, there were an estimated total 134 000 SCD related hospitalizations accounting for an estimated $811 million dollars. This hospitalization frequency includes a 30% re-admission rate within 30 days.^5^

Multiple factors contribute to the high number of hospitalizations and emergency department (ED) visits for individuals with SCD, which notably includes inconsistent access to outpatient primary care clinicians (PCCs). In a recent national survey of 1060 family practice physicians, only 20% reported being comfortable with overall management of SCD.^6^ However, 80% of respondents indicated they would be willing to co-manage pediatric patients, and 68% reported they would be willing to co-manage adult patients. Importantly, 69% of respondents perceived clinical decision support tools would be useful in their practice to help guide the treatment of patients with SCD. However, few resources have historically been available for PCCs to assist with the management of SCD.^6^

Another important factor leading to hospitalization is management strategies utilized by EDs and identification of individuals requiring close follow up. It has been established that rapid aggressive pain control is more likely to reduce pain to a level that can be managed at home and avoid hospital admission. Recent data demonstrated that for every 10-minute increase in the delay to treat VOC pain in the ED, the risk of hospital admission increased by 4.7%.^7^ In 2018, Tanabe et al reported use of individualized versus a weight-based analgesic protocol resulted in lower hospitalization rates.^8^ In addition, systematic identification of high-risk patients, or patients with a large number of ED visits may benefit from the implementation of a care management referral program, which led to an increase in outpatient care management services delivered.^9^

Therefore, we conducted a dissemination project in the state of North Carolina (NC). We adapted the National Heart Lung and Blood Institute (NHLBI)^10^ recommendations for the treatment of SCD to user friendly algorithms and health maintenance plans (decision support tools) that were included in a mobile application (app), the “SCD Toolbox” (developed by the team).^11^ The app, as well as paper versions of the tools, were available for distribution in person and accessible on multiple websites. We disseminated the toolkit to clinicians throughout NC with the goal to reduce costs and increase utilization of primary care practices for patients with SCD. We used the Community Care of North Carolina (CCNC) network, comprised of 14 different geographic networks to disseminate the tools to PCCs. We also disseminated these decision support tools to ED providers throughout NC. In this paper we: 1) report the dissemination strategy; 2) examine the effects of the decision support tools on study outcomes (ED visits, inpatient hospitalizations, outpatient care by provider type, and HU and opioid prescription fills) during the pre- and post- dissemination time periods; and 3) conduct a cost- savings analysis of the dissemination of the decision support tools and its effect on health-care resource utilization. The cost-savings analysis is meant to measure the extent to which the financial benefits associated with the dissemination of the SCD toolkit surpassed the costs of dissemination. We hypothesized that our toolkit of evidence-based SCD guidelines would decrease health-care costs and increase outpatient utilization of health-care resources in NC.

## METHODS

### Design

A pre-post dissemination strategy was used to conduct the project. The project was approved by the Duke University Institutional Review Board and waiver of consent was granted.

### Partners and Decision Support Tool Development

#### Primary Care and Pediatrician Tools

The study team participated in a task force led by ME, the deputy chief medical officer at CCNC, NC’s previous, single managed care solution. Team members included PCCs, pediatric and emergency medicine providers from this project as well as other expert clinicians affiliated with CCNC. The 2014 NHLBI “Recommendations for the Management of Sickle Cell Disease” were adapted for dissemination to PCCs and pediatric providers including: health maintenance charts (adults and children); HU monitoring (adults and children); anemia, fever (adults and children); respiratory symptoms; neurological symptoms; and VOC. These decision support tools are available at https://www.communitycarenc.org/what-we-do/clinical-programs/pediatrics/tools/sickle-cell-disease, and http://sickleemergency.duke.edu/frontpage.

#### ED Tools: Treatment of VOC and Referral for Care Management

A separate CCNC task force was led by PT (emergency nurse scientist) and included members of CCNC and the President of the North Carolina College of Emergency Physicians (NCCEP). The task force adapted the VOC recommendations and developed an algorithm for the treatment of VOC in the ED. The algorithm is included in the SCD Toolbox and located on the following website: (https://sickleemergency.duke.edu/pain-and-case-management). In addition to recommendations for rapid, aggressive pain management, this algorithm incorporates disposition decision recommendations, as well as a recommendation to screen all patients for psychosocial needs and refer all SCD patients to their CCNC affiliated care manager. A link to the succinct screening tool is included in the algorithm and on the website. The VOC algorithm was formally endorsed by the North Carolina Emergency Nurses Association (NC-ENA) as well as NCCEP.

#### Toolbox Availability

The health maintenance charts and algorithms for PCCs and pediatric and emergency medicine providers were made available as part of the toolbox, including paper versions that could be disseminated in person, or made available on a website. An app, the SCD Toolbox, was developed including an iPhone and Android version and is available in the app Store.

### Dissemination of the Toolbox

Separate dissemination strategies were used for distribution to primary care, pediatric and ED providers.

#### PCC and Pediatric Providers

We partnered with CCNC to disseminate tools to PCCs and pediatricians in NC. At the time of the project, CCNC was NC’s Medicaid managed care approach with a network of 1800 PCC practices in NC, accounting for 90% of pediatricians and family medicine and internal medicine providers in the state.^12^ CCNC, which manages 1.4 million enrollees (70% of whom are children), is a physician-led, community-based organization that created 14 regional networks in the state to carry out population health initiatives.^13^ CCNC has infrastructure in place to support PCCs and coach them to improve quality and efficiency. Each network has a team of care managers and quality improvement (QI) specialists and QI leads who target individuals who are at-risk for poor outcomes or high health-care utilization. CCNC uses care management and informatics to help defragment the fragmented health-care system in NC. CCNC has achieved substantial cost savings by emphasizing clinical initiatives that have an impact on cost and population health. CCNC has saved over $1 billion over a 4-year period and exceeds national benchmarks in diabetes, and asthma care.^14,15^

A train-the-trainer model was used to disseminate the toolbox to PCCs that participated in CCNC. The investigator team developed and delivered a presentation (via recorded webinar) that was delivered on several occasions to the QI leads for each of the networks. Each QI lead was then accountable to deliver the presentation and disseminate the toolbox to their QI specialists for practices within their network that had a minimum of five SCD patients within their practice. Over time, most of the QI specialists reported disseminating the toolbox to all practices with SCD patients. Dissemination began in February 2018 for most of the networks and concluded in September 2018. We were unable to track actual dissemination to the providers from the QI specialists.

To further support dissemination to PCCs, we worked with the NC Department of Health and Human Services (DHHS), the NC Academy of Family Physicians (NCAFP), and the NC Pediatric Society (NC PEDs). DHHS wrote a letter of support that was posted on our research website: https://sickleaware.nursing.duke.edu/section-content/provider-information. The NCAFP and NC PEDs distributed the link, along with a brief description about the SCD Toolbox in an email blast and e-newsletter to their members. NCAFP has approximately 4000 members total (2800 practicing doctors, 300 residents, 200 retirees, and 800 medical students) and NC PEDs has about 2300 active members—about 1500 pediatricians plus retirees, residents, mid-levels, staff and others.

#### Emergency Providers

We provided 16 face-to-face in-services to seven EDs throughout NC. These EDs were associated with the highest number of ED visits for the treatment of SCD. Presentations were given by the study investigators who were either an experienced ED physician or nurse. Presentations were given to nurses, attending and resident physicians, nurse practitioners (NPs) and physician assistants (PAs). During the course of the project, we met monthly with leadership of the NC-ENA to identify state and local chapter meetings in which our team was invited to provide short presentations on the toolkit. We also provided four brief educational sessions at NC-ENA Chapter meetings. Finally, both the NC-ENA and the NC College of Emergency Physicians posted the link to the toolkit on their respective websites. Over the course of the dissemination period, we directly disseminated the VOC algorithm and case management referral form to 90 ED physicians, 274 ED nurses, two case managers and three ED educators.^16^ We were unable to capture how many ED physicians and nurses accessed the toolkit through our website.

### Data Sources and Sample

We used Medicaid claims data provided by CCNC for March 2016 through August 2019; CCNC received all Medicaid claims data for NC at the time of this project. The data were extracted in five waves and included claims from SCD patients including HbSS, HbSC, and HbS-thalassemia, excluding sickle-cell trait (ICD 10 CM codes: D57.0x, D57.1, D57.2x, D57.8x).

Medicaid State Drug Utilization Data is included to measure aggregate Medicaid expenditures on Endari, a prescription drug approved in 2018, to manage SCD.

In addition to the CCNC Medicaid data, we also used salary information from Duke University, the Bureau of Labor and Statistics, and Medscape to estimate the time costs of individuals participating in the toolkit dissemination training.

#### Sample

The sample was limited to those who were enrolled in Medicaid for the entire sample wave. Dual Medicare/Medicaid eligible enrollees were excluded within each sample wave. This exclusion ensured that all relevant expenditures were captured in our dataset and did not accrue to another payor besides Medicaid. We also excluded outlier expenditure enrollee-month observations, defined as enrollee months with total expenditures greater than the 99th percentile of expenditures observed in our sample, or about $8063.

#### SCD Toolkit Implementation Dates

We recorded the date each network implemented the SCD toolkit and analyzed the data examining the network dissemination date with a two-month run-in period. Dates of implementation by CCNC network site ranged from February 2018 to January 2019 and are included in Supplementary Table 1. Implementation dates were matched to claims data based on the CCNC network.

#### Variables

We examined utilization and expenditures across seven categories: primary care, outpatient, ED, hematology, inpatient, NPs, PAs, and two prescription claims, HU and opioids. These categories were chosen because they comprise almost 98% of total spending and because these are the service categories where any effect of the dissemination was likely to occur.

#### Utilization and Prescription Refill Variables

For utilization, we examined monthly visits for each of the visit-related categories. These data were structured as count data by monthly visits. For prescriptions, we examined HU and opioid refills, defined by examining the number of medication refills by month. Since claims data was used, we were unable to measure medication possession ratio or proportion of days covered.

#### Cost-Savings Analysis Variables

The cost-savings analysis estimates both total costs and total savings of toolkit dissemination. The estimated total cost of the dissemination is the dollar cost of producing the relevant toolkit training materials, conducting toolkit training sessions, and the costs for the CCNC networks to further disseminate information from the toolkit training sessions to the relevant staff for both the primary care and ED settings. For the cost of producing the relevant toolkit training materials, we used the time spent on creating materials by Duke faculty and the per-hour labor cost for the associated individuals based on salary information from Duke. For the cost of conducting the toolkit training sessions, we estimated the time costs for Duke personnel as well as time costs for individuals attending the toolkit training sessions. For Duke personnel, we estimated the time spent on scheduling and conducting the toolkit training sessions and their per-hour labor costs. For non-CCNC individuals attending the toolkit training sessions, we counted person-hours by category based on the number of attendees and length of toolkit training and multiplied the hours for each category by an estimate of the per-hour labor cost using salary information from the Bureau of Labor and Statistics (BLS)^17^ or Medscape^18^ for physicians, nurses, and care managers. Nurse costs per hour were derived from annual mean wage from the BLS for 29-1141, or “Registered Nurses,” in NC. Case manager costs per hour were derived from annual mean wage from the BLS for 29-0000, or “Health-care Support Workers, All Other,” in NC. The case manager annual mean wage from the BLS ($34 400) was similar to the reported average salary for “Hospital Case Managers” reported on Glassdoor ($36 000) for the same year. The emergency medicine physician costs per hour were derived from the annual compensation for the Mid-Atlantic from slide 7 of “Medscape EM Physician Compensation Report 2015”^18^ and converted to 2019 dollars with the medical care consumer price index. Salaries were converted to per-hour costs assuming 2080 working hours per year. The costs for CCNC network staff to attend the sessions and further disseminate information from the toolkit training sessions were taken from the CCNC budgets for these activities from each individual network obtained directly from CCNC. The estimated cost of dissemination by activity type and setting is available in Supplementary Table 2.

The total savings are measured by estimating the reduction in the per-enrollee per-month (PEPM) Medicaid expenditures that occurred after the SCD toolkit training. Similar to the utilization variables, we analyzed expenditures by categories (primary care, outpatient, ED, hematology, inpatient, NPs, PAs, and two prescription claims, HU and opioids) as well as total expenditures.

#### Control Variables

Patient age was recorded as the age first reported in the claims data. Sex represents the participant’s first reported instance of sex (male/female). We created variables for rural or metro residential location using claim zip codes. Additionally, we created a variable for length of CCNC enrollment calculated by the amount of time enrollees spent in the CCNC network.

Aggregate Medicaid expenditures on Endari prescriptions are included to control for potential confounding of the estimated effect on the SCD toolkit training dissemination. Lastly, we created a quarterly dummy variable to account for seasonality of the claims and possible correlation with the outcomes of interest.

### Statistical Methods

We analyzed the data using STATA 16.1. We examined the change in outcomes (utilization, prescription refills, and expenditures) before and after implementation of the SCD toolkit. The Medicaid data was examined monthly for each outcome over the study period. No data were missing, as such, it was appropriate to analyze the data using complete case analysis. First, we examined the data descriptively, examining patients’ claims’ characteristics and outcomes pre-implementation and post-implementation of the SCD toolkit. We also examined utilization, prescription refills, and expenditures using an interrupted time series (ITS) regression model with a trend-change-only specification. We chose a trend-change-only specification for ITS because the effect of the toolkit training dissemination was expected to occur gradually and not result in a discrete change in PEPM expenditures at the time of dissemination. In the ITS model, we controlled for clinically significant patient characteristics (age, gender, county rural or metro status), as well as Endari spending. Network fixed effects are included to control for time-invariant regional differences in expenditures. We control for seasonality by including a quarterly indicator. The regression model was run separately by category. For analyzing expenditures, we also ran the model for total expenditures. Total expenditures included all expenditures incurred by each enrollee in each month, not only those represented in the nine service categories we analyzed separately. The utilization outcomes were displayed using an incidence rate ratio calculated with a negative binomial distribution, as appropriate for count data, while the cost-savings analysis outcomes were displayed with a coefficient for general linear regression. All outcomes used the ITS model and clinically significant control variables. Significance was set at 0.05 for all outcomes.

#### Additional Utilization Statistical Analysis

To further assess the association between outcomes and exposures of interest for utilization and prescription outcomes, we assessed the number of visits by month using Generalized Linear Models applying Generalized Estimating Equations (GEE) for count data to evaluate whether the utilization rates differed significantly across time by exposure (pre- and post-implementation of the SCD toolkit). Since we determined the data were over-dispersed, the final model used the negative binomial distribution for over-dispersion. In this final model, we accounted for the Medicaid enrollee (patient ID) and month of claim (time variable). While we discussed the results of both the ITS and GEE approach for utilization and prescription refills, the tables focus on the ITS results. This allows for comparability and clinical interpretation between the utilization analysis and cost-savings analysis.

## RESULTS

### Characteristics of Medicaid Enrollees by Month

**Figure 1.**
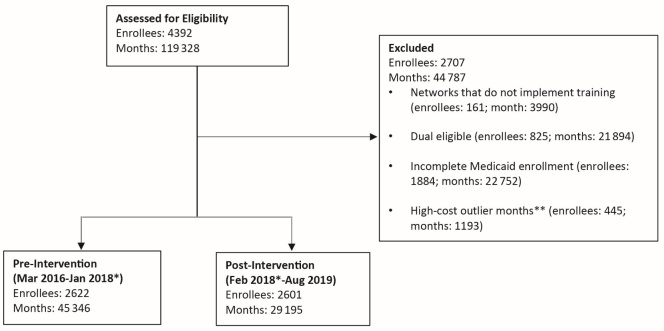
Sample Exclusions * Post-intervention dates vary by CCNC network. The first network to implement the SCD toolkit training dissemination was in February 2018. By June 2018, 10 of the 12 participating networks had implemented the training dissemination. The final network implemented the training in January 2019. ** High-cost outliers are defined as total expenditures exceeding the 99th percentile for a given enrollee-month. Medicaid enrollment is measured as the total number of months in a sample wave where an enrollee has Medicaid coverage. We exclude all enrollee-months within a sample wave (either 12 or 6 months) if the total Medicaid enrolled months are less than the total sample wave months. Our final modeling sample includes 3135 unique enrollees. We see 66.4% of enrollees across both pre- and post- intervention periods. Seventeen percent of enrollees are observed in the pre-period only, and 16.4% are observed in the post-period only. Enrollee counts across pre-intervention, post-intervention, and excluded categories do not sum to total due to overlap (enrollees may appear in both the pre-intervention and post-intervention, as well as having certain months excluded). The enrollee months across pre-intervention, post-intervention, and excluded categories do sum to the total.

From 2016-2019, the CCNC network contained 4392 enrollees diagnosed with SCD; of the enrollees, 119 328 months were represented in the sample. Given the study question, networks that did not implement toolkit training, had enrollees who were also eligible for Medicare, had enrollees with incomplete Medicaid enrollment, and had high-cost outlier months were excluded from the cohort (Figure 1). Of the remaining 3135 enrollees, who represent a total of 74 541 months, included in the final cohort; 45 346 months were included pre-intervention, while 29 195 months were included post-intervention. The mean age of the cohort was 17.99±13.45 years, 32 623 (43.8%) were male, and 59 003 (79.2%) resided in a Metro location (Table 1). There were no observed descriptive differences in age, sex, and residential location pre- and post-intervention (Table 1).

**Table 1.** Summary Statistics for Enrollee Characteristics Pre- and Post-Intervention

	**Total Claims**	**Pre-Intervention**	**Post-Intervention**
	**N=74 541**	**n=45 346**	**n=29 195**
**Patient Claims Characteristics**			
**Sex, n (%)**			
Female	41 918 (56.2%)	25 678 (56.6%)	16 240 (55.6%)
Male	32 623 (43.8%)	19 668 (43.4%)	12 955 (44.4%)
**Age, mean (SD)**	17.99 (13.45)	18.21 (13.45)	17.66 (13.45)
**Age, median (IQR)**	15.00 (8.00-25.00)	15.00 (8.00-25.00)	15.00 (7.00-25.00)
**CCNC program months enrolled, mean (SD)**	0.93 (0.23)	0.93 (0.22)	0.92 (0.25)
**Median (IQR)**	1.00 (1.00-1.00)	1.00 (1.00-1.00)	1.00 (1.00-1.00)
**Aggregate Monthly Spending on Endari**			
**Average Monthly Endari Spending ($1000s), mean (SD)**	31.10 (34.37)	6.46 (17.41)	69.38 (11.69)
**Average Monthly Endari Spending ($1000s), median (IQR)**	10.01 (0.00-70.15)	0.00 (0.00-0.00)	70.58 (70.15- 78.27)
**Residence Claims Characteristics**			
**Residence, n (%)**			
Metro	59 003 (79.2%)	35 981 (79.3%)	23 022 (78.9%)
Non-Metro adjacent to metro	13 553 (18.2%)	8271 (18.2%)	5282 (18.1%)
Non-Metro un-adjacent to metro	1985 (2.7%)	1094 (2.4%)	891 (3.1%)
**CCNC network, n (%)**			
1	14 341 (19.2%)	8499 (18.7%)	5842 (20.0%)
2	6400 (8.6%)	3547 (7.8%)	2853 (9.8%)
3	5083 (6.8%)	2937 (6.5%)	2146 (7.4%)
4	2759 (3.7%)	2311 (5.1%)	448 (1.5%)
5	4234 (5.7%)	2815 (6.2%)	1419 (4.9%)
6	3463 (4.6%)	1970 (4.3%)	1493 (5.1%)
7	8339 (11.2%)	4777 (10.5%)	3562 (12.2%)
8	686 (0.9%)	406 (0.9%)	280 (1.0%)
9	12 519 (16.8%)	6951 (15.3%)	5568 (19.1%)
10	5007 (6.7%)	3197 (7.1%)	1810 (6.2%)
11	5193 (7.0%)	3925 (8.7%)	1268 (4.3%)
12	6517 (8.7%)	4011 (8.8%)	2506 (8.6%)

Abbreviations: CCNC, Community Care of North Carolina; IQR, interquartile range; SD, standard deviation.

Missing data: Age – none, CCNC months enrolled – none, gender - none, rural – none, CCNC network - none.

Abbreviations for CCNC Network: Access East, 1; Access Care, 2; Carolina Collaborative Community Care, 3; Community Care of Southern Piedmont, 4; Community Care of the Lower Cape Fear, 5; Community Care of the Sandhills, 6; Community Care of Wake/Johnston Counties, 7; Community Care of Western North Carolina, 8; Community Care Partners of Greater Mecklenburg, 9; North Piedmont Community Care, 10; Northwest Community Care Network, 11; Partnership for Community Care, 12.

### Utilization and Prescription Refill Claims by Month

There were a total of 65 046 utilization claims across the categories of interest, 39 847 pre-intervention and 25 199 post- intervention, or 0.88 and 0.86 claims per enrollee per month on average, respectively. Of these, 17 404 were primary care visits, representing the largest number of claims, while 16 316 were ED visits with the second largest total claims. During the study timeframe, there were 14 818 HU refills and 14 659 opioid refills (Table 2).

**Table 2.** Description of Monthly Utilization and Cost Outcome Variables Pre- and Post-Intervention

	**Total Monthly All**	**Total Monthly Pre- Intervention**	**Total Monthly Post- Intervention**	**Mean Per Month All**	**Mean Per Month Pre- Intervention**	**Mean Per Month Post- Intervention**
**Utilization Outcomes**						
**Primary care visit (n)**	17 404	10 751	6653	0.23 (0.54)	0.24 (0.55)	0.23 (0.53)
**Hospital outpatient visit (n)**	8214	5289	2925	0.11 (0.41)	0.12 (0.42)	0.10 (0.40)
**Emergency department visit (n)**	16 316	9993	6323	0.22 (0.67)	0.22 (0.68)	0.22 (0.66)
**Hematology visit (n)**	7194	4655	2539	0.10 (0.34)	0.10 (0.36)	0.09 (0.31)
**Inpatient hospitalization (n)**	6231	3832	2399	0.08 (0.30)	0.08 (0.31)	0.08 (0.30)
**Nurse practitioner visit (n)**	5800	2920	2880	0.08 (0.31)	0.06 (0.27)	0.10 (0.35)
**Physician assistant visit (n)**	3887	2407	1480	0.05 (0.25)	0.05 (0.25)	0.05 (0.24)
**Total**	65 046	39 847	25 199	0.87 (1.32)	0.88 (1.32)	0.86 (1.31)
**Prescription Outcomes**						
**Hydroxyurea fill (n)**	14 818	9025	5793	0.20 (0.43)	0.20 (0.43)	0.20 (0.43)
**Opioid fill (n)**	14 659	9694	4965	0.20 (0.53)	0.21 (0.55)	0.17 (0.51)
**Total**	29 477	18 719	10 758	0.40 (0.70)	0.41 (0.71)	0.37 (0.69)
**Expenditure Outcomes**						
**Primary care visit (mean)**	1 805 354	1 076 916	728 438	24.22 (100.16)	23.75 (101.53)	24.95 (98.01)
**Hospital outpatient visit (mean)**	1 768 466	1 126 117	642 348	23.72 (170.62)	24.83 (174.77)	22.00 (163.94)
**Emergency department visit (mean)**	8 453 860	5 079 877	3 373 983	113.41 (414.11)	112.02 (407.80)	115.57 (423.73)
**Hematology visit (mean)**	625 145	404 129	221 017	8.39 (33.40)	8.91 (35.70)	7.57 (29.45)
**Inpatient hospitalization (mean)**	20 286 910	12 721 319	7 565 591	272.16 (1037.95)	280.54 (1067.65)	259.14 (989.94)
**Nurse practitioner visit (mean)**	449 763	230 848	218 915	6.03 (26.08)	5.09 (24.25)	7.50 (28.64)
**Physician assistant visit (mean)**	314 320	191 557	122 764	4.22 (27.41)	4.22 (29.50)	4.20 (23.81)
**Hydroxyurea fill (mean)**	460 015	296 987	163 027	6.17 (17.35)	6.55 (19.06)	5.58 (14.26)
**Opioid fill (mean)**	1 196 954	792 157	404 797	16.06 (106.75)	17.47 (107.56)	13.87 (105.46)
**Total (mean)**	36 538 539	22 632 360	13 906 179	490.18 (1230.54)	499.10 (1250.70)	476.32 (1198.45)

Missing: None; Utilization data: Total counts reflect the total number of visits per outcome pre- and post-intervention. Mean statistics show the mean number of visits per enrollee and month pre- and post-intervention. Cost data: Total expenditures included in each outcome category for all enrollees in each sample period. Statistics based on sample from regression analysis, which includes 75 541 enrollee months and is described in the Data Sources and Sample section.

When examined in a GEE model controlling for potentially confounding variables, there was a 12% observed decreased rate of primary care visits per enrollee-month after implementation of the SCD toolkit (Incidence Risk Ratio [IRR]: 0.88 (0.81-0.97), P=0.008). This decrease in primary care visits was further confirmed when examining primary care visits in a time series model controlling for relevant variables (pre-intervention trend, IRR: 1.00 [95% confidence interval (CI): 0.99-1.00] vs post-intervention change in trend, IRR: 0.99 [95% CI: 0.98-0.99], P<0.001). The clinical significance of this is unknown.

There was a trend of increased visits to NPs pre-intervention (IRR: 1.04 [95% CI: 1.02-1.07], P=0.003), but that trend was no longer seen in the post-intervention change in trend (IRR: 0.98 [95% CI: 0.95-1.00], P=0.074). Additionally, there was an observed change in opioid refills with a significant increase in opioid refills observed post-intervention (pre-intervention trend IRR: -0.99 [95% CI: 0.99-0.98], P=0.005 and post-intervention change in trend, IRR: 1.01 [95% CI: 1.00-1.02], P=0.006) (Table 3). The clinical significance of this is unclear. There were no observed significant changes in inpatient hospitalizations, hospital outpatient, ED, hematology, NP or PA visits, or in HU refills with implementation of the SCD toolkit (Table 3).

**Table 3.** Interrupted Time Series Regression Results for Primary Outcomes of Interest

	**Pre-Intervention Trend**		**Post-Intervention Change in Trend**	
**N=74 532**	**IRR (95% CI)**	***P*-value**	**IRR (95% CI)**	***P*-value**
**Utilization Outcomes**				
**Primary care visit**	1.00 (0.99-1.00)	0.168	0.99 (0.98-0.99)	<0.001
**Hospital outpatient visit**	1.00 (0.98-1.04)	0.530	0.97 (0.94-1.01)	0.101
**Emergency department visit**	1.00 (0.99-1.01)	0.750	1.00 (0.98-1.02)	0.915
**Hematology visit**	1.00 (0.98-1.01)	0.563	1.00 (0.97-1.03)	0.973
**Inpatient hospitalization**	1.00 (1.00-1.01)	0.261	1.00 (0.98-1.01)	0.306
**Nurse practitioner visit**	1.04 (1.02-1.07)	0.003	0.98 (0.95-1.00)	0.074
**Physician assistant visit**	1.02 (0.99-1.05)	0.171	0.98 (0.95-1.00)	0.058
**Prescription Utilization Outcomes**				
**Hydroxyurea fill**	1.00 (1.00-1.01)	0.056	1.00 (1.00-1.01)	0.637
**Opioid fill**	0.99 (0.99-0.98)	0.005	1.01 (1.00-1.02)	0.006
				
	**Coef. (95% CI)**	***P*-value**	**Coef. (95% CI)**	***P*-value**
**Expenditure Outcomes**				
**Primary care**	0.05 (-0.08, 0.18)	0.450	-0.24 (-0.66, 0.17)	0.220
**Hospital outpatient**	0.3 (-0.32, 0.93)	0.310	-0.6 (-1.46, 0.26)	0.160
**Emergency department**	0.65 (-0.76, 2.07)	0.330	-0.83 (-3.31, 1.66)	0.480
**Hematology**	-0.06 (-0.25, 0.12)	0.460	0.13 (-0.16, 0.42)	0.340
**Inpatient hospitalization**	0.12 (-2.09, 2.34)	0.900	-0.26 (-3.09, 2.58)	0.850
**Nurse practitioner**	0.24 (0.09, 0.39)	0.000	-0.08 (-0.24, 0.07)	0.260
**Physician assistant**	0.08 (-0.05, 0.21)	0.190	-0.09 (-0.23, 0.05)	0.190
**Prescription Expenditure Outcomes**				
**Hydroxyurea**	-0.05 (-0.12, 0.01)	0.070	0.07 (-0.05, 0.19)	0.240
**Opioid**	0.06 (-0.25, 0.37)	0.670	0.01 (-0.20, 0.23)	0.910
**Total Expenditures**				
**Total**	1.35 (-1.68, 4.39)	0.35	-1.71 (-5.30, 1.88)	0.32

Abbreviations: IRR, incidence risk ratio; CI, confidence interval; Community Care of North Carolina, CCNC.

* Interrupted time series analysis linearly displaying change in utilization and expenditures over time. The Pre-Intervention Trend column reflects the monthly trend in the pre-intervention period before the dissemination was implemented. The Post-Intervention Change in Trend column reflects the change in the estimated trend caused by the dissemination implementation. The model accounts for time, dissemination of the toolkit, controlling for age, age squared, rural/metro location, gender, Endari spending, network enrollment, seasonality, and network site. Cost outliers are excluded. Results for utilization display an IRR and 95% CI in parenthesis using a negative binomial distribution as appropriate. Results for expenditures display the coefficient and the 95% CI. Significance is set at P<0.05. Standard errors are clustered at the CCNC network level.

### Cost-Savings Analysis

There was a trend toward increased expenditures for NPs pre-intervention ($0.24 PEPM increase) and the SCD toolkit dissemination was not associated with a statistically significant change to this trend in the post-intervention period. There was also no observed significant change in total expenditures, but the change in the trend for the post-intervention period is negative.

**Figure 2.**
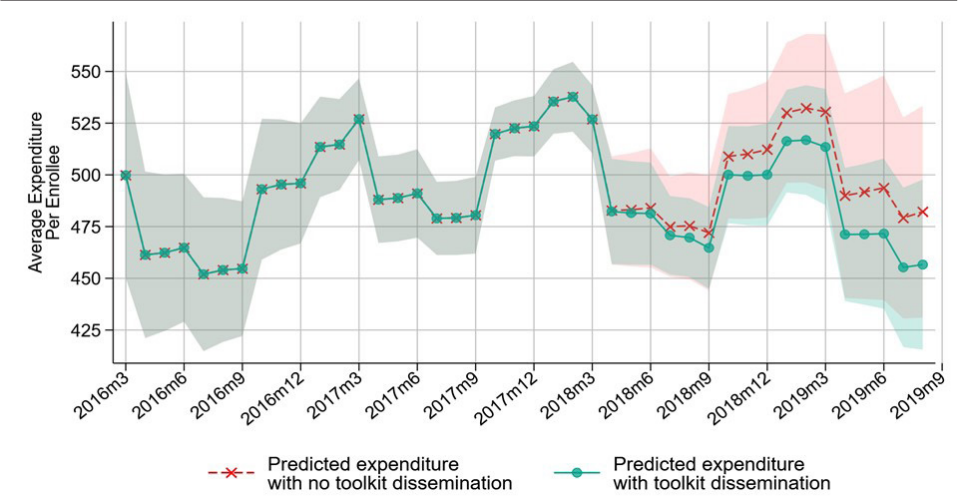
Estimated Change in Total Expenditures after SCD Toolkit Dissemination Abbreviations: CCNC, Community Care of North Carolina; PEPM, per-enrollee per-month; SCD, sickle cell disease. Predicted expenditures with no toolkit dissemination, shown in red X’s, represent the predicted PEPM expenditures with the SCD training dissemination indicator set to zero in all months. Predicted expenditures with toolkit dissemination, shown in green circles, represent predicted PEPM expenditures with the actual toolkit training dissemination timing. As CCNC networks begin disseminating the training, we estimate a decline in PEPM expenditures. The shaded areas represent 95% confidence intervals.

Although estimated effects are not statistically significant, we provide calculations of the implied total savings to assess the economic significance of the dissemination of the SCD toolkit. The estimated benefits are calculated by comparing predicted PEPM spending with a counterfactual scenario where no dissemination implementation occurs (based on the pre-period implementation trend in expenditures) and multiplying the differential PEPM expenditures by the number of enrollee months in the post-intervention period. The estimated financial benefit of the SCD toolkit dissemination overall was an estimated $409 503 of savings in Medicaid expenditures (Table 4). The cost of disseminating the SCD toolkit was estimated at $48 089, yielding a net cost savings of $361 414. While the calculated net cost savings is large, we have not identified evidence of net cost savings or net benefit of the dissemination since the estimated financial benefit was not statistically significant. Figure 2 shows the predicted expenditures over time with and without SCD toolkit dissemination. While the results from the cost-savings analysis were not statistically significant, directionally they are suggestive that health-care expenditures declined after dissemination.

**Table 4.** Total Estimated Expenditure Benefit after Dissemination

	**Total Estimated Benefits**	**Standard Error**	**95% CI**	
**Primary care**	-58 515	44 773	-146 270	29 240
**Hospital outpatient**	-142 569	93 551	-325 929	40 791
**Emergency department**	-198 124	270 025	-727 373	331 125
**Hematology**	31 526	31 498	-30 210	93 262
**Inpatient hospitalization**	-61 062	308 147	-665 030	542 906
**Nurse practitioner**	-20 325	17 085	-53 812	13 162
**Physician assistant**	-21 895	15 515	-52 304	8514
**Hydroxyurea**	16 703	13 503	-9763	43 169
**Opioid**	2606	23 431	-43 319	48 531
**Total**	-409 503	390 142	-1 174 181	355 175

Abbreviations: CI, confidence interval; PEPM, per-enrollee per-month; SCD, sickle cell disease.

* Estimated benefits implied by the SCD toolkit dissemination are calculated by comparing predicted PEPM spending with a counterfactual scenario where no dissemination implementation occurs. Estimated benefits (cost reductions) are presented as negative values. All dollars are in constant 2019 U.S. dollars adjusted using the medical care consumer price index. The sum total of all sub-categories does not add the total estimated benefits because we have not included every possible sub-category that is incorporated into total costs.

### Sensitivity Analyses

We also analyzed the cost savings in regressions with and without excluding outlier member months for total expenditures, with and without excluding individuals who are eligible for both Medicaid and Medicare, and with and without the two- month run in period for the timing of the post-intervention period. In each version of the model, we found similar overall savings levels that were not statistically significant.

## DISCUSSION

Our team embarked upon a statewide dissemination project with an analysis of cost savings. The RE-AIM (reach, effectiveness, adoption, implementation and maintenance) framework is a common implementation science model used to guide program implementation.^19,20^ “Reach” focuses on maximizing the likelihood that the intervention or program being disseminated reaches the maximum number of stakeholders who will use the intervention. Without excellent reach, it is impossible to understand the effectiveness, adoption, implementation, and maintenance of the intervention. As this project focused only on dissemination, understanding the reach was critical to our success. We established strong partnerships with multiple disciplines, state health agencies, and professional organizations to maximize our reach. These relationships allowed us to work collaboratively with multiple stakeholders across NC to both adapt the NHLBI recommendations into user-friendly formats of the SCD toolbox, and disseminate the toolbox throughout NC. The development of the toolbox and formation of these collaborations were quite successful. Dissemination to EDs was more successful than to PCCs and pediatricians. We previously reported 29%-42% of ED provider survey respondents reported awareness of the toolbox and the VOC algorithm, with success measured via electronically administered surveys.^16^ Our dissemination to PCCs and pediatricians was more difficult. PCC dissemination was affected by the re-organization of CCNC, which began at the beginning of the project and continued throughout; this resulted in a major challenge to optimal dissemination. At the start of the dissemination, CCNC functioned as the only true managed care organization in NC. Subsequently, legislation was passed that required a transition to multiple managed care organizations in NC; ultimately five managed care organizations were established. This resulted in turnover of the QI specialists and leads that were responsible for dissemination of the toolbox to the providers in their network. We were able to track dissemination of the training; 14 networks received training including dissemination to a total of 41 practices. While we are confident dissemination occurred, we are not able to truly capture the true success, or reach of the dissemination to the actual providers. We know that dissemination was often to the practice administrator who may or may not have disseminated to the providers. Finally, for both PCC and ED providers, systematizing the recommendations within an electronic health record has been found to improve uptake of guidelines, and was explicitly expressed by ED providers when surveyed.^21^

The hypothesized shift from ED and inpatient health care use was unrealized, and in the opposite direction as we found a minor decrease in primary care visits, although this had a wide CI. We did not find a change in HU fills, but an increase in opioid refills was noted post-intervention. However, the meaning of these findings are unclear and could be attributed to many factors. The decrease in PCP visits and increase in opioid prescriptions may be related. Of note, this project was implemented during the opioid epidemic. In 2017, NC implemented the Stop Act to limit opioid prescribing. Patients with SCD require opioids for the treatment of both acute and chronic pain. With the Stop Act, it became more difficult for patients with SCD to obtain these medications. It is possible that alternatively, patients had more visits with pain or other specialists who may have been providing these opioids; it is clear patients did receive more opioids during this time, despite the Stop Act. If this were the case, with better pain control it is possible they did not seek as many PCP visits. It is also possible that because we were unable to track which PCPs received the intervention, the PCPs who received the intervention actually did prescribe more opioids as suggested by the pain algorithm. Anecdotally, we did identify PCCs who felt more confident co-managing their patients with SCD, particularly those providers with a small number of SCD patients in their practice.

Finally, while we did not find a statistically significant cost savings to dissemination of the toolkit, the data suggest a trend in savings over time that was economically significant given the magnitude relative to the costs. Our post-intervention follow-up analysis time was relatively short. With additional time, and opportunities to reinforce the intervention, we believe it is likely that this intervention could result in cost savings. Further, our estimate of the savings is based on an intent to treat (ITT) estimation since we do not know how many or which health-care providers ultimately received information on the toolkit. Because of the ITT approach, we are not estimating the average treatment effect of implementing the toolkit, which would be higher than the ITT estimate if compliance of implementing the toolkit was anything less than 100%. The use of the toolbox by providers may actually have resulted in costs savings. However, due to the inability to track use by provider, we cannot state this with confidence. Moving forward, most of the cost associated with the toolkit has now already been completed. Other states or practice settings that wish to disseminate the toolbox would not have to incur these “start-up” costs. The primary cost associated with a dissemination project would include provider or staff time to disseminate the intervention. Costs can be minimized by using professional organizations, state health organizations, or health systems to disseminate via electronic media or at standing conferences or meetings.

### Limitations

There are several limitations to the project. We included only individuals with Medicaid in NC. Nationally, it is estimated that 70% of individuals with SCD have Medicaid. It is possible that individuals with private insurance may have incurred higher expenses. Some individuals with SCD are also dual eligible for Medicaid and Medicare; those individuals were excluded from this study and could also have incurred higher costs. It is also possible that insurers may have different limits or requirements for reimbursing for ED visits and hospitalizations, which may also contribute to a difference in costs. Another limitation is we did not have data for any patients with SCD who were not potentially affected by the dissemination and thus did not have a comparison group to control for general trends in expenditures relevant for this population. We also did not have any risk scores or diagnosis data to account for underlying shifts in the health of the population that may have been occurring during this period. True implementation of a large scale requires a longer time frame to evaluate long-term effects, as well as booster sessions and audit and feedback to providers. Future analysis should examine differences in cost by patient utilization type (high vs. low) and age. An important limitation of the project was being unable to track exactly whether a provider in the practice actually received the toolbox; we used an ITT model for analysis and are aware practices received the toolkit but the provider may not have received it. Finally, we did not measure any benefits outside of direct medical costs such as mortality or quality-adjusted life years saved. We are unable to identify decedents in the sample and the criteria for full Medicaid enrollment likely excludes any decedents from the sample. Thus, our estimation results do not capture any effect on either mortality or related expenditures incurred at the end of life.

## CONCLUSIONS

Our team successfully adapted evidence-based recommendations and created a toolbox of algorithms and health maintenance charts guiding treatment of individuals with SCD. We disseminated the toolbox, including an app, to primary care, pediatric, and ED providers throughout NC. Due to many challenges with the dissemination of the toolbox, the anticipated shift from ED visits and hospitalizations to better outpatient management did not occur. However, a trend toward cost savings did occur. Future dissemination should include systematizing guidelines within electronic health records, establishing local provider champions, and leveraging state health officials, professional associations, and health system leaders.

## Supplementary Material

Supplementary Material
