# Supplementary material for: Dissemination of Evidence-Based Recommendations for Sickle Cell Disease to Primary Care and Emergency Department Providers in North Carolina: A Cost Benefit Analysis
Source: J Health Econ Outcomes Res. 2021 Apr 1;8(1):18–28. doi: 10.36469/jheor.2021.21535 (PMC8016663; doi:10.36469/jheor.2021.21535)
Supplement: Supplementary Material [file jheor_2021_8_1_21535_56507.pdf]

Journal of Health Economics and Outcomes Research

# Supplementary Online Material

Tanabe P, Blewer A, Bonnabeau E, et al. Dissemination of evidence-based recommendations for sickle cell disease to primary care and emergency department providers in North Carolina: a cost benefit analysis. *JHEOR*. 2021;8(1):18-28.
doi:10.36469/jheor.2021.21535

**Supplementary Table 1.** Dissemination Timing Across Networks (Networks #4 and #11 Did Not Implement Toolkit Dissemination Training)

**Supplementary Table 2.** Total Estimated Cost of Toolkit Dissemination

This supplementary material has been provided by the authors to give readers additional information about their work.

This is an open-access article distributed under the terms of the Creative Commons Attribution 4.0 International License (CCBY-4.0). View this license's legal deed at http://creativecommons.org/licenses/by/4.0 and legal code at http://creativecommons.org/licenses/by/4.0/legalcode for more information.

**Supplementary Table 1. Dissemination Timing Across Networks (Networks #4 and #11 Did Not Implement Toolkit Dissemination Training)**

| CCNC Network | Date |
|---|---|
| 1 | Mar-18 |
| 2 | Feb-18 |
| 3 | Apr-18 |
| 5 | Jan-19 |
| 6 | Jun-18 |
| 7 | Feb-18 |
| 8 | Mar-18 |
| 9 | Mar-18 |
| 10 | Feb-18 |
| 12 | May-18 |
| 13 | Sep-18 |
| 14 | May-18 |

Abbreviations: Community Care North Carolina, CCNC

**Supplementary Table 2. Total Estimated Cost of Toolkit Dissemination**

| Activity | Total Estimated Cost ($) |
|---|---|
| **Development of primary care materials** | 472 |
| **Scheduling primary care sessions** | 517 |
| **Conducting primary care sessions** | 1589 |
| **CCNC cost of attending sessions for primary care & further toolkit dissemination** | 27 600 |
| | |
| **Development of ED materials** | 472 |
| **Scheduling ED sessions** | 671 |
| **Conducting ED sessions** | 3195 |
| **Attending Sessions for ED** | 13 574 |
| | |
| **Total Dissemination Costs** | 48 089 |

Abbreviations: emergency department; ED.

*For the cost of producing the relevant toolkit training materials, we used the time spent on creating materials by Duke faculty and the per-hour labor cost for the associated individuals based on salary information from Duke. For the cost of conducting the toolkit training sessions, we estimated the time costs for Duke personnel as well as time costs for individuals attending the toolkit training sessions. All dollars are in constant 2019 U.S. dollars adjusted using the medical care consumer price index. The sub-category costs do not add up to the total costs due to rounding.
